# The Spectrum of Major Seed Storage Genes and Proteins in Oats (*Avena sativa*)

**DOI:** 10.1371/journal.pone.0083569

**Published:** 2014-07-23

**Authors:** Olin D. Anderson

**Affiliations:** Agricultural Research Service, United States Department of Agriculture, Albany, California, United States of America; Universidad Miguel Hernández de Elche, Spain

## Abstract

**Background:**

The oat seed storage proteins are mainly composed of two classes: the globulins and avenins. Among the major cereals, the globulins are the major seed protein class in rice and oats, and along with the higher protein content of oats is the basis for the relative higher nutrition content in oats compared to the other cereals. The second major class of oat seed proteins is the avenins; also classified as prolamins – seed proteins high in proline and glutamine amino acids. The prolamins are associated with celiac disease, an autoimmune disorder of the gastrointestinal tract. In spite of their importance, neither the oat globulins nor the avenins have been completely analyzed and described for any single germplasm.

**Results:**

Using available EST resources for a single hexaploid oat cultivar, the spectrum of avenin and globulin sequences are described for the gene coding regions and the derived protein sequences. The nine unique avenin sequences are suggested to be divided into 3–4 distinct subclasses distributed in the hexaploid genome. The globulins from the same germplasm include 24 distinct sequences. Variation in globulin size results mainly from a glutamine-rich domain, similar to as in the avenins, and to variation in the C-terminal sequence domain. Two globulin genes have premature stop codons that shorten the resulting polypeptides by 9 and 17 amino acids, and eight of the globulin sequences form a branch of the globulins not previously reported.

**Conclusions:**

A more complete description of the major oat seed proteins should allow a more thorough analysis of their contributions to those oat seed characteristics related to nutritional value, evolutionary history, and celiac disease association.

## Introduction

The plant seed proteins provide reserves for the development of the seedling after germination. Most cereal seed proteins belong to one of two classes; i.e., the globulins (salt-soluble proteins) and the prolamins (alcohol soluble proteins). The globulins are the major protein seed component for oats (*Avena sativa*) and rice, while the prolamins are the predominate form in maize, sorghum, and the Triticeae tribe (wheat, barley, rye) [1]. In oats, the globulins comprise 50–80% of the seed protein and the prolamins 10–20% [2]. Purported advantages of oats over other cereals are features such as being anticholesterolaemic, antioxidant, diuretic, and possessing increased fiber [3]. In addition, since the globulins have a more favorable composition of essential amino acids, and since oat seeds have a higher protein content than rice, oats are generally considered to possess higher basic nutritional quality than the other major cereals [4]. However, the prolamin component of the oat seed, the avenins, shares the basic feature of other cereal prolamins as being polypeptides relatively rich in proline and glutamine. It is these regions of the amino acid sequences that are associated with the elicitation of celiac disease, an autoimmune disorder of the gastrointestinal tract [5]. Although oats are considered generally a safer alternative to other cereals where the prolamins are the dominant seed protein (wheat, barley, rye, maize, sorghum), it is not clear what are the existence and prevalence of celiac eliciting epitopes in the oat avenins [6]. In addition, while many celiac patients evidently tolerate including oats in their diets, at least some persons can show mucosal inflammation when exposed to avenins from at least some oat varieties [7].

The oat globulins exist in the seed as hexamers of approximately 320,000 Da – each subunit composed of a 54,000 Da polypeptide disulfide crosslinked to a 32,000 Da polypeptide [8]. The two subunit polypeptides are originally joined as a single translated polypeptide that is post-translational cleaved. In contrast, the avenins have been variously reported in the 17,000–34,000 Da range [9], [10], and can exist both as monomers and disulfide linked aggregates [6].

Several estimates have been made of the number of oat avenin and globulin genes and proteins. Using the translation products of seed mRNA (messenger RNA), it has been reported that there are at least 20 distinct globulin large subunits and 10 small subunits as resolved by 2D polyacrylamide electrophoresis (PAGE) [8]. One possible explanation in the different number of large and small subunits would be if the small subunits are too similar in sequence and properties to be easily distinguished by PAGE. Two other estimates of oat globulin gene numbers utilized Southern blot analysis and estimated either 7–10 globulin genes [11] or 50 globulin genes [12] per haploid genome. For the avenins, different techniques with different oat germplasms find at least 11 total avenin polypeptide components in the 20–34 Da range [12], 25 genes per haploid genome using Southern blot analysis [13], and eleven genes by EST (expressed sequence tag) analysis [14].

Despite reasons for interest in the two major classes of oat seed proteins, there is not a comprehensive description of either the oat globulins or avenins. Single, or a few, sequences have been reported from individual oat cultivars [6], [8], [10], [11], [13], [15], [16]. There is a report of a number of avenin DNA sequences, but they were from different germplasms and thus not representative of complete oat avenin gene families [6]. There are also a large number of avenin DNA sequences in Genbank, but not formally reported or analyzed in the literature, that have originated from both a variety of *A. sativa* cultivars and from other species of *Avena*. These latter sequences were generated via PCR and represent an unknown coverage of the total avenin family. A recent report states, without details in the analysis or giving sequences, that there were eleven different avenin sequences found within the ESTs of a single oat cultivar [14]. For the oat globulins, there has as yet been no complete oat globulin sequence family. Missing for both the oat avenins and globulins is such a detailed and comprehensive description.

Although the best method of identifying complete gene sets is using a high-quality complete genome sequence, no such resource is currently available for oats, nor is likely to be available for some time. As a substitute, if sufficient high-quality ESTs are available, sets of active gene coding region sequences can be assembled. It is also best to use ESTs from single germplasms to avoid the complications of sorting out allelic sequences and differing gene set compositions.

In the current report, ESTs for the oat cultivar CDC Dancer are used to assemble a proposed complete set of active oat avenin and globulin seed protein gene coding and derived amino acid sequences. Nine unique sequences are identified for the avenins and 24 for the globulins. The sequences of both classes generally agree with previously reported sequences. In addition, novel classes are reported. The composition and structure of the sequence families are analyzed and discussed along with evolutionary aspects of the families, relative representation of sequences in the EST resource, and issues in nomenclature within the grass prolamins.

## Materials and Methods

### EST assembly

A total of 17,776 ESTs for the oat cultivar (cv) CDC Dancer were downloaded from GenBank. A description of the CDC Dancer EST generation can be accessed at the GrainGenes database site [17]. All CDC Dancer ESTs are from the seed. ESTs were assembled with the Seqman module of the Lasergene suite (DNAstar, Inc.). All resulting contigs composed of three or more ESTs were annotated for the closest matching sequence of monocots. ESTs of contigs annotated as avenins or globulins were merged for subsequent assembly and analysis. There are also 7,097 oat ESTs from cv Ogle in GenBank, but are from a range of tissues and do not include enough seed ESTs to attempt seed protein sequence assemblies.

### Avenin ESTs

After contig annotation, a total of 351 ESTs were identified as matching avenin sequences. Experience has shown that a reiterative process is necessary for prolamin EST assembly – likely due to the similar sequences within these gene families and the repetitive regions within their sequences. For oat avenins, ESTs were assembled starting with only ESTs greater than 600 bases long. Experience has shown that longer ESTs create more reliable initial scaffolds for prolamin assemblies. Additional ESTs were than added to the assembly sequentially; i.e., ESTs 500–600 bases, 400–500, 300–400, and 200–300. ESTs with coding sequences shorter than 200 bases were discarded. Individual contigs were examined for potential chimeric ESTs causing a truncation in contig assembly. Suspected chimeras were checked by BLAST [18] analysis and ESTs with 5′ or 3′ sequence not matching avenins were removed. In cases where most of the EST sequence was UTR (untranslated region), the remaining coding sequence was too short for reliable assembling and such ESTs were also removed. The remaining ESTs were reassembled and the resulting contigs were divided into classes according to having five or more ESTs or fewer than five. The former were assembled together to find overlapping sequences not initially joined by the software. The resulting contigs were individually assembled with ESTs from the contigs with fewer than five ESTs and those matching exactly were merged into the larger contigs. Finally, each larger contig was manually inspected for ESTs apparently not matching the contig. These were removed and compared to the other contig consensus sequences and merged if found a match – otherwise were left as unassigned.

To check if ESTs might have been assigned incorrectly, possibly due to sequence similarity among avenin gene family members, the ESTs assigned to each final contig were assembled with the other eight contigs’ consensus sequences. No EST matched with any final contig except the contig to which each EST was assigned. All ESTs assigned to specific oat cultivar CDC Dancer avenin and globulin contigs are given in Table S1.

The junction of the signal peptides and mature polypeptides for both avenins and globulins was determined by comparison of derived amino acid sequences from the DNA contigs to sequenced oat proteins and related proteins from other cereals.

### Globulin ESTs

From the total CDC Dancer ESTs, 887 were identified as globulin sequences and reassembled. As with the avenin ESTs, assemblies can often be improved with sequential assemblies. The globulin reassembly started with EST of 600 bp of longer, then adding the remaining ESTs longer than 500 bp, 400 bp, 300 bp, and finally 250 bp. Preliminary analysis had indicated some globulins being almost identical in sequence and therefore globulin ESTs with coding sequences shorter than 250 bp were not used. Resulting contigs with single ESTs 5′ or 3′ extensions were checked for chimeric ESTs which were removed and the contig reassembled as with the avenin ESTs.

### Phylogenetic analysis

Comparisons of sequences was using MEGA 5.10 [19] and ClustalW for alignments and the Neighbor-joining algorithm for phylogenetic tree generation. Validity of the generated trees were checked by bootstrap with 500 iterations. Alignments were checked manually and adjusted as needed – particularly within repetitive regions where alignment algorithms have difficulty with prolamins.

## Results

### Avenins

A total of 324 ESTs from cv CDC Dancer were identified as avenin sequences and remained after removing potential chimeras and ESTs with only short regions of coding sequence. After the 324 ESTs were reassembled, 311 ESTs were assigned to nine unique contigs (File S1). The nine CDC Dancer avenins are named with the protein class, cultivar, and number; i.e., Avenin-CDCDancer-1. For the current report, the origin from cv CDC Dancer is understood; e.g., Avenin-1.

Thirteen ESTs remained unassigned and unique. It is assumed that most of these are aberrant sequences resulting from one of the common problems known in cloning, amplifying, and sequencing prolamin DNA sequences; i.e., deletions in repetitive sections and chimeras generated from two or more avenin sequences.

**Figure 1:**
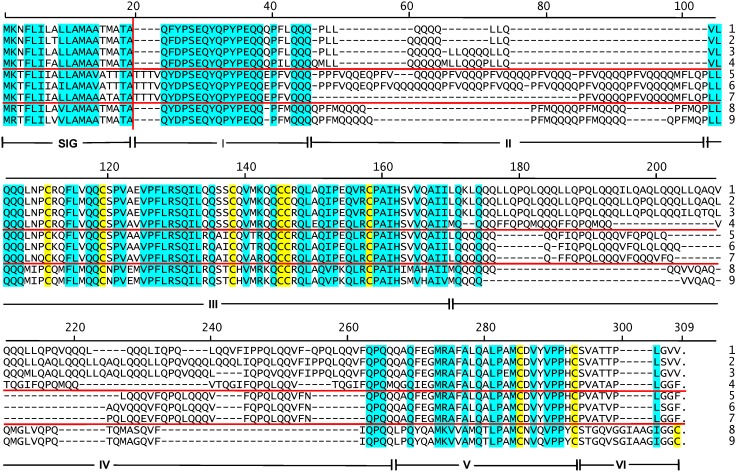
Alignment of avenin amino acid sequences. The derived amino acid sequences of the nine cv CDC Dancer avenins are aligned with ClustalW. Positions within the total alignment are noted above the sequences. Domains of the polypeptides are indicated below the sequences in Roman numerals. Amino acid residue positions conserved in all nine sequences are shaded blue. Conservation being defined as the same amino acid or amino acids with similar characteristics; i.e., D/E, F/Y, K/R, L/I/V/A, S/T. Cysteine residues are shaded yellow. Horizontal red lines separate major subclasses. The vertical red line indicates the site of cleavage of the signal peptide during post-translational processing.

**Figure 2:**
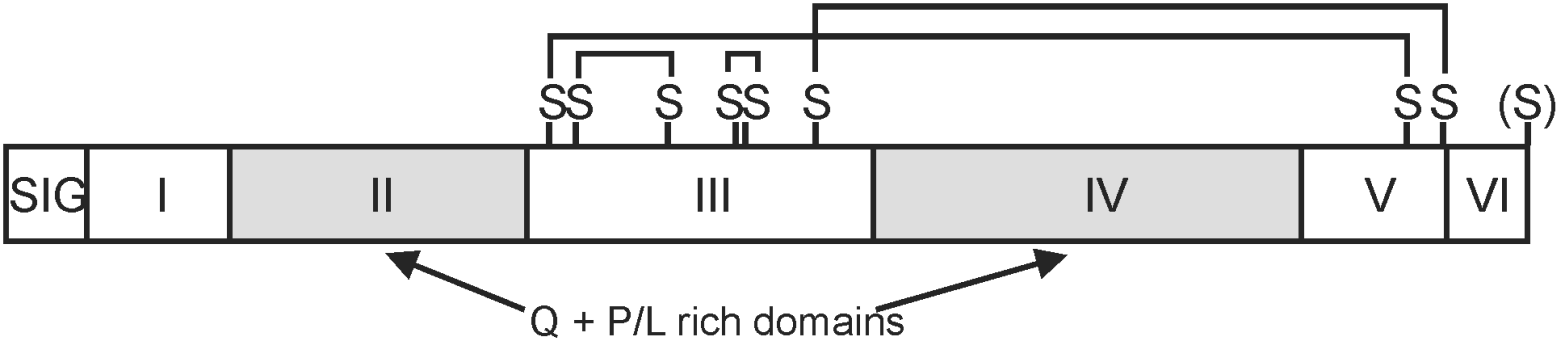
Model of oat avenin polypeptide organization. The avenin polypeptide structure is shown divided into regions labeled by Roman numerals (I to VI). The signal peptide is SIG. Two glutamine plus proline/leucine regions are indicated and shaded. Cysteine residue positions are indicated by an S (representing the cysteine sulfhydryl group) and lines connecting the cysteines are intramolecular bonds previously determined for an oat avenin [16]. (S) represents the additional cysteine in some avenins.

Since avenin genes do not have introns, the coding regions defined by the EST contigs also define the gene sequence from the start to stop codons. The nine derived amino acid sequences are given in File S2 and aligned as shown in Figure 1 where the numbered positions above the alignments refer to the alignment and not to any specific polypeptide. After processing off of the 19 amino acid signal peptide (vertical red line in Figure 1), the nine coding regions encode polypeptides that range from 190 to 255 amino acids and 21,913 to 28,342 Da (Table 1). The nine avenins have the typical high proline + glutamine content of prolamins; i.e., 39.4–47.6%. The most conserved amino acid residue positions are shaded blue in Figure 1 and do not contain most of the proline and glutamine amino acid residues which are concentrated mainly in two unshaded regions of the polypeptides. It is proposed to consider the structure of these avenins to be composed of seven domains (Figures 1–2). The first domain is the signal peptide (SIG) cleaved during protein processing. This is followed by six domains of the mature avenin. The first domain (I) is a 22–26 residue relatively conserved sequence followed by a domain (II) especially high in glutamine plus a number of proline and leucine residues. Domain III is 75 residue positions of conserved sequence followed by a second domain (IV) high in glutamine, proline, and leucine. The C-terminal portion of the avenins includes a third conserved domain (V) of 24 residues and the polypeptide ending with 10 or 15 non-conserved amino acid residues (VI).

**Table 1:** Oat cv CDC Dancer avenin and globulin protein characteristics.

Protein	Length^1^	Daltons^2^	%Proline	%Glutamine	%Pro+Glu	No. ESTs^3^
Avenin-1	232	25,861	9.7	35.4	45.1	61
Avenin-2	234	27,148	9.4	37.2	46.6	62
Avenin-3	255	27,448	8.6	34.1	42.7	50
Avenin-4	201	23.276	10.0	31.8	41.8	16
Avenin-5	236	27,599	11.9	35.2	47.1	14
Avenin-6	242	28,342	11.2	36.4	47.6	23
Avenin-7	242	28,342	11.2	36.4	47.6	59
Avenin-8	192	22,182	10.4	29.7	40.1	11
Avenin-9	190	21,913	10.5	28.9	39.4	10
Glo-1	479	54,332	5.0	12.7	17.7	15
Glo-2	479	54,112	5.2	13.2	18.4	39
Glo-3	479	54,343	5.0	13.6	18.6	34
Glo-4	478	54,195	4.8	13.0	17.8	20
Glo-5	479	54,211	4.8	12.9	17.7	37
Glo-7	486	54,995	5.3	13.6	18.9	17
Glo-8	478	54,335	5.0	11.7	16.7	8
Glo-9	490	55,628	4.9	12.9	17.8	36
Glo-10	488	55,379	4.3	14.1	18.4	37
Glo-11	518	55,917	4.6	13.3	17.9	28
Glo-12	494	55,841	5.1	14.0	19.1	47
Glo-13	494	55,865	4.9	14.0	18.9	34
Glo-14	488	55,356	4.3	13.7	18.0	18
Glo-15	477	53,911	4.6	13.6	18.2	12
Glo-16	503	56,575	4.6	13.9	18.5	28
Glo-23	491	55,634	4.9	14.1	19.0	147

1 Number of amino acid residues in mature polypeptide.

2 Calcultated Daltons to the first stop codon.

3 Number of assigned ESTs in contig.

The intramolecular disulfide bonds of an avenin similar to Avenins 5–7 have been previously reported [12] and the pattern of bonds is assumed to be similar in all other avenins (Figure 2). These eight conserved cysteine residues that form four intramolecular disulfide bridges are typical of portions of the basic pattern of the AAI_LTSS (alpha-amylase-inhibitor, lipid transfer, seed storage) protein superfamily [20]. Two of the avenins, Avenin-8 and Avenin-9, have a ninth cysteine as their C-terminal amino acid – presumably available for intermolecular disulfide bonds.

The nine CDC Dancer avenin sequences were also compared to other known full-length *A. sativa* avenin protein sequences and found to cluster well with branches including the other 67 sequences from a variety of *A. sativa* germplasms (Figure S1). This result indicates no additional major subclasses of active avenins genes likely exist in cv CDC Dancer – with the variations in sequence indicated in Figure S1 being consistent with variation among the different alleles and complements from different germplasms.

**Figure 3:**
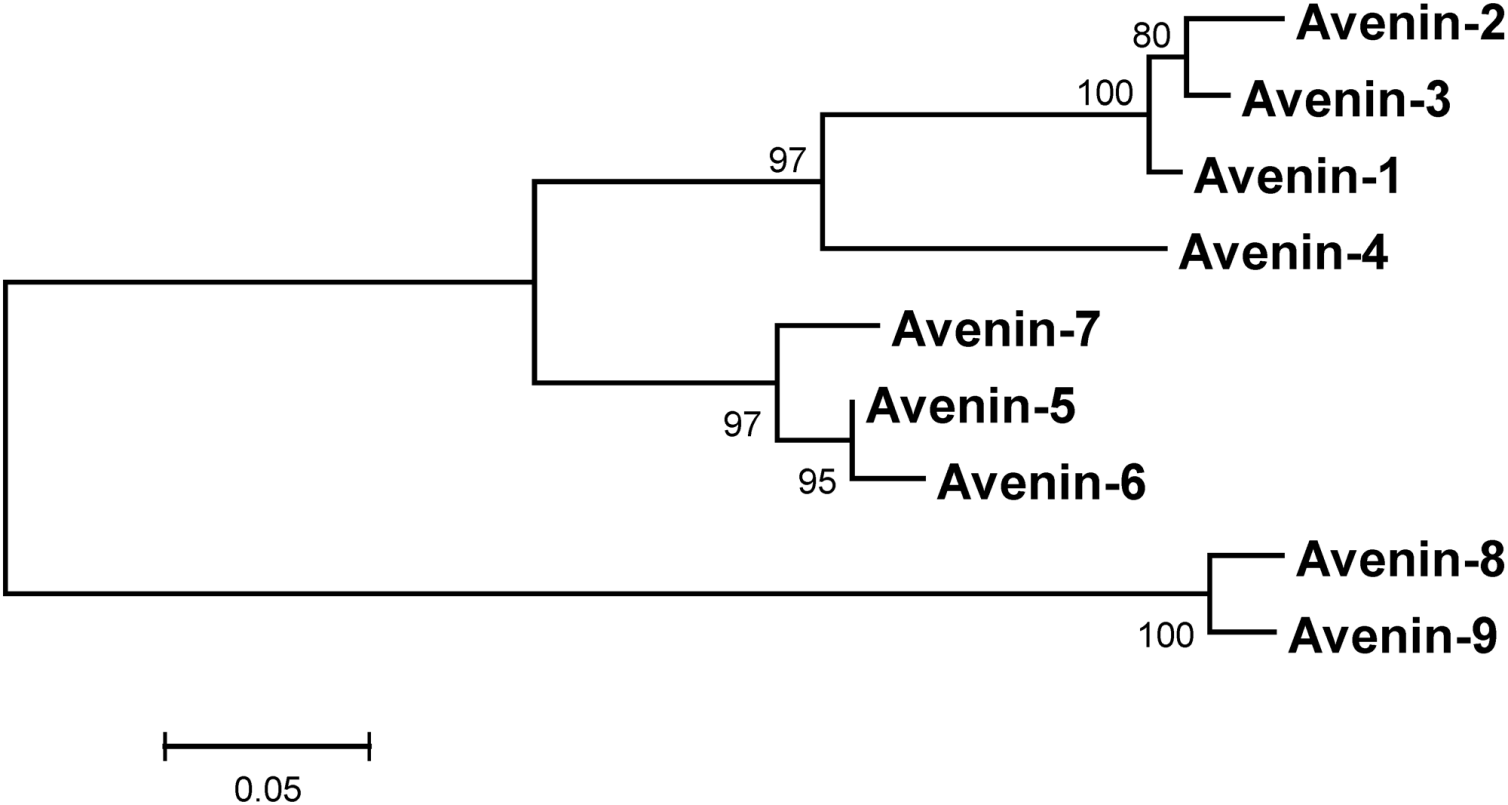
Phylogenetic tree of CDC Dancer avenins. The avenin amino acid alignment in Figure 1 is used to generate a phylogenetic tree by the Neighbor-joining algorithm. Tree validity was checked by bootstrap using 500 iterations.

The alignment of CDC Dancer avenins was used to generate a phylogenetic tree (Figure 3) whose branches suggest three groupings; i.e., Avenins 1–4, 6–7, and 8–9. Unique features of these groupings can be seen where red horizontal lines separate the three groupings (Figure 1). Distinguishing features of the three groupings include mature Avenins 5–7 beginning with an additional four amino acids (TTTV) compared to the other two groupings, Avenins 8–9 having five additional amino acids in domain VI, and the three different surrounding residue contexts for cysteine-3 at position 138 (SSCQ, AECQ, STCH).

Although results clearly indicate at least three main branches, there is evidence that Avenin-4 may belong in either its own branch or at least counted as a variant of the top branch. Note that of the nine avenins, Avenin-4 both is more distantly branched from the other avenins than the others within their clusters (1–3, 6–7, 8–9). The amino acid sequence of Avenin-4 shows variations from Avenins 1–3, particularly in the two variable regions. A phylogenetic tree of the nine CDC Dancer avenins with the 67 other currently available full-length avenin amino acid sequences from different germplasms shows clustering of Avenin-4 with 12 other avenin sequences in such a distinct branch (Figure S1).

### Avenin Repetitive domains

**Figure 4:**
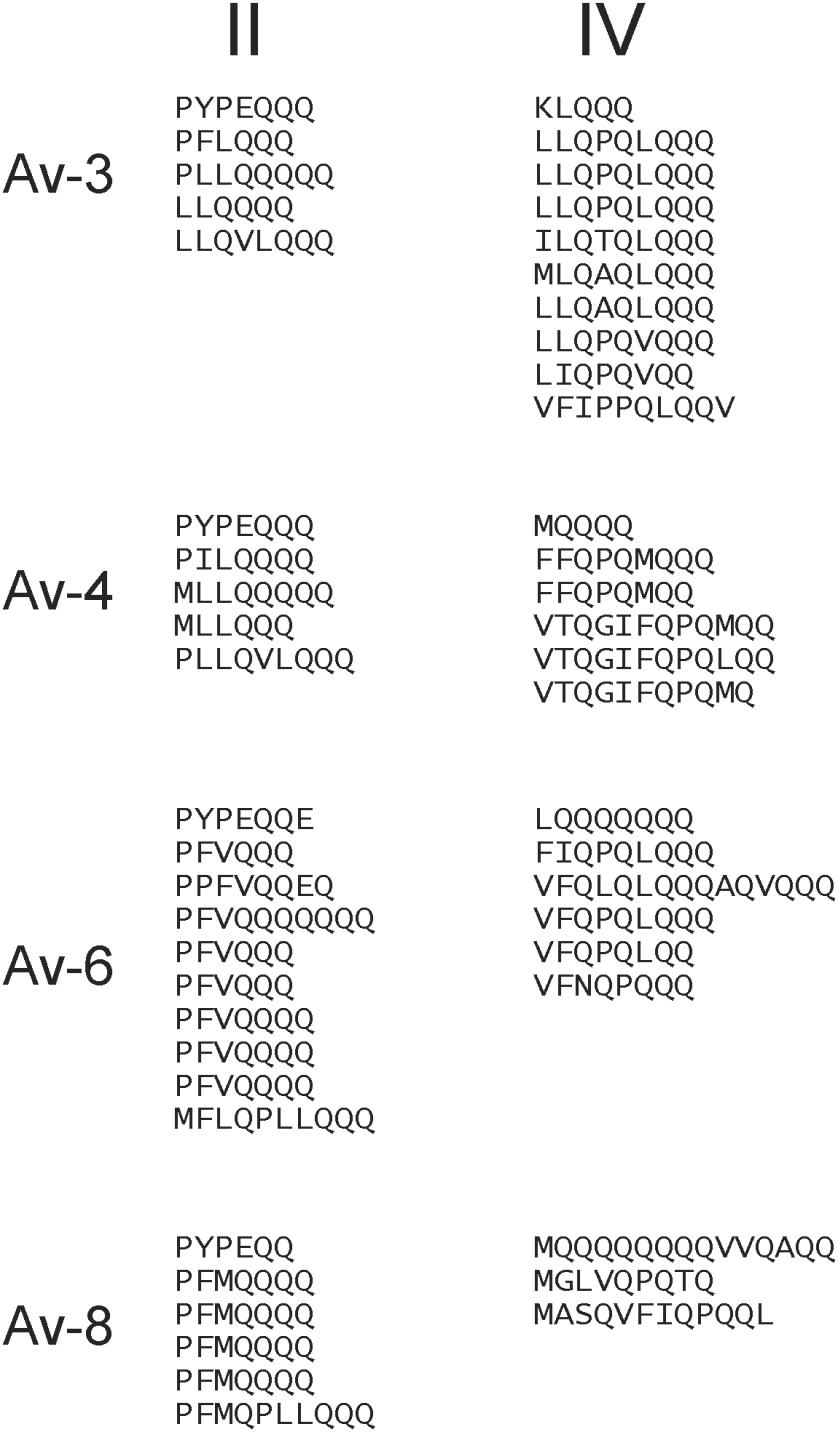
Avenin repetitive units. The repetitive units for domains II and IV are arrayed vertically for four of the CDC Dancer avenins.

Most of the variation in avenin sequence length occurs in domains II and IV – the two regions rich in glutamine plus proline/leucine. In the Triticeae prolamins, there is a single high proline + glutamine domain that is composed of variations on simple motif units somewhat characteristic of each prolamin type. A similar pattern can be seen in the domains II and IV of the oat avenins. The amino acid sequences of the two proline + glutamine rich domains of four avenins from the main branches can be arrayed vertically to emphasize proposed repeat units (Figure 4). Domain II is composed of motifs with a predominant PF(V/L/M)Q_3–7_ pattern. There is less confidence in proposing a common motif pattern for domain IV among the different avenin subclasses, although Avenins 3, 4, and 6 have predominant patterns within each avenin subclass. Note that the repeats do not exactly coincide with the domain borders since the first and last repeats are among conserved sequences and thus placed in the conserved domains (Figure 1).

It is also noted that the alignment emphasizes that the variable domains (II and IV) are evolving faster than the rest of the sequence (domains I, III, V, VI). Caution is required in considered phylogenetic analyses that include prolamin repetitive/high−P+Q regions. To check if the avenin phylogenetic tree branching pattern (Figure 3) was influenced by inclusion of the two variable regions, those sequence sections were removed from the nine CDC Dancer avenins and the alignment and phylogenetic tree generated were both repeated. The branching pattern was exactly the same (not shown).

### Globulins

**Figure 5:**
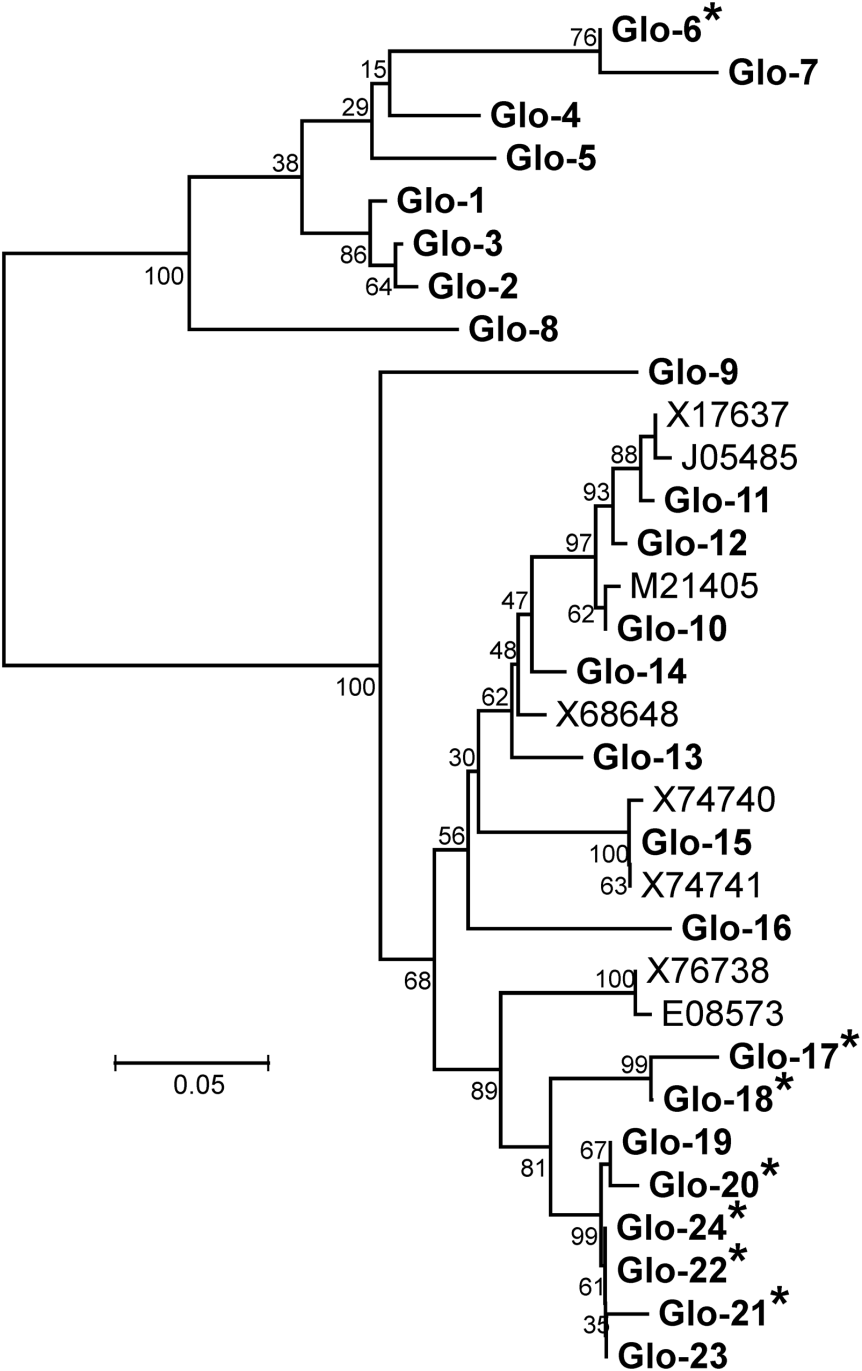
Phylogenetic tree of CDC Dancer globulins. Shown is a phylogenetic tree oat avenin amino acid sequences constructed by the Neighbor-joining algorithm from a ClustalW alignment of all 24 CDC Dancer globulins (in bold) and the eight oat globulin sequences currently available in Genbank (labeled by accession numbers). Astericks indicate partial sequences. Tree validity was checked by bootstrap using 500 iterations.

A similar procedure was used to assemble oat seed globulin EST sequences as was used with the avenins. After removing sequences shorter than 250 bp and chimeras, 692 ESTs assembled into 24 unique globulin sequences (named Glo-1 to Glo-24). Sixteen of these encode full-length polypeptides and eight encode truncated sequences (all consensus DNA sequences are found in File S3). The DNA sequences were used to derive the amino acid sequences (File S4) and aligned and displayed in a phylogenetic tree along with the eight previously reported oat globulin entries in Genbank (Figure 5).

Seven of the eight truncated sequences are missing their C-terminal coding portion: Glo-17 to Glo-22 and Glo-24. All seven cluster closely in a branch with Glo-23 – the only full-length sequence in that branch (Figure 5). Of the 247 ESTs assigned to sequences in this branch, 149 form the Glo-23 contig. The alignment of the derived amino acid sequences of Glo-19 to Glo-24 finds only six amino acid differences among the six sequences, but all are supported by multiple ESTs (not shown). Glo-22, -23, and -24 are have identical amino acid sequences – the differences in their positions in the phylogenetic tree due to their different lengths. When examining the DNA coding sequences of those three, they only differ in one base position in all three pairwise combinations (not shown). Similarly, the Glo-19 and Glo-20 DNA sequences differ from each other by three bases, and both differ from Glo-23 by four bases. The most likely explanation for the number of truncated sequence EST contigs in this branch, considering the similarity in the determined unique sequences, is that ESTs encoding the C-termini of the avenins in this branch have identical sequences and the assembly software, unable to distinguish among the individual sequences, simply places all the C-terminal encoding ESTs in the largest contig; i.e., that for Avenin-23.

**Figure 6:**
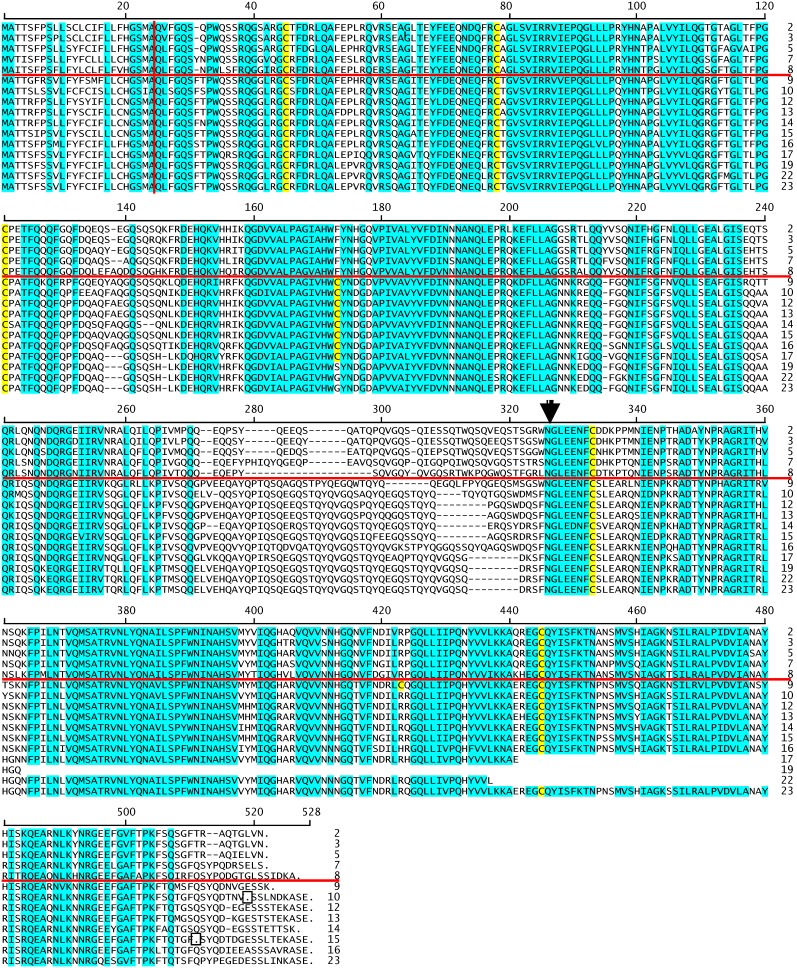
Alignment of oat globulin amino acid sequences. Sixteen of the CDC Dancer globulin derived amino acid sequences are aligned by ClustalW. Sequences are labeled by number only; i.e., “2” indicates sequence Glo-2. Not shown are eight sequences that are either C-terminal truncated or almost identical to shown sequences. The horizontal red line separates two main classes of oat globulins. The vertical red line indicates the end of the signal peptide. Amino acid residue positions conserved in all sixteen sequences are shaded blue. Conservation is defined as the same amino acid or amino acids with similar characteristics; i.e., D/E, F/Y, K/R, L/I/V/A, S/T. Cysteine residues are shaded yellow. The arrowhead following position 326 indicates site of the post-translational cleavage generating two disulfide crosslinked polypeptides. Premature stop codons are indicated by boxed periods in Glo-10 and Glo-15.

Likewise, Glo-17 and Glo-18 are similar each other and close to the Glo-19 to Glo-24 grouping. Since Glo-17 only covers the N-terminal half of a full-length globulin sequence it can as yet only be speculated that similar 3′ coding sequences lead to ESTs being assigned incorrectly. The lack of 3′ sequence for some globulin sequences, and the speculation that the cause is identical gene sequences in this region, is consistent with the report of fewer than expected unique globulin small subunits [8] since that 3′ region encodes the small subunit (junction shown as arrowhead in Figure 6).

The eighth non-full-length sequence is Glo-6, with Glo-7 the most similar sequence. Glo-6 is missing the 5′ 1/3 of a full-length sequence and is the only 5′ truncated sequence. In this case, it is likely either the 5′ end of the gene is so similar to other globulins that the ESTs went to other contigs, and/or a lack of ESTs in this region for this gene.

### Comparison of oat globulins

Sixteen of the oat globulin sequences encoded full-length globulin proteins. The first observation was that the previously reported sequences did not include one of the two main branches of oat cv CDC Dancer globulins i.e., Glo-1 to Glo-8 (Figure 5). Table 1 gives the length in amino acids of the full-length mature globulins, the calculated Daltons, the percentages of glutamine and proline residues, and the number of assigned ESTs. The sixteen polypeptide sequences range from 477 to 518 amino acid residues and Da from 53,911 to 56,575 Da. In contrast to the avenin proline + glutamine content of 39.4–47.6%, the oat globulins are 16.7–19.1%. An alignment was made of sixteen of the oat globulin amino acid sequences that were chosen to show the most variation in sequence (Figure 6) – minus five of the truncated sequences (globulins 6, 18, 20–21, 24) and globulins with little variation in sequence (Figure 5; globulins 1, 4, 11). In this amino acid alignment, the vertical red line indicates the end of the signal peptide and the horizontal red line separates the two main branches of sequences (Figure 5). Regions of high conservation are indicated by blue shading and cysteine residues by yellow shading. The arrowhead indicates where post-translational cleavage separates the original complete polypeptide into the acidic and basic polypeptides [11].

The mature oat globulins have two main regions of sequence difference. The first region is part of a domain (237–325) reminiscent of the avenin variable regions in being relatively high in glutamines. The first part of this domain shows some conservation, while the remainder (257–325) is of more variable length and composition. For example, within positions 237–325 of Glo-23 there are 26 glutamine out of 81 residues. The remainder contains only two prolines, but also 15 neutral residues (A, G, I, L, V) and 14 hydroxyl-group residues (S, T, Y). In some of the globulins this region has discernible repetitive pattern over part of the region; e.g., Glo-23 has 32 amino acids at positions 282–315 that can be aligned as four octamers of QYQE/VGQST (not shown). Other globulins, such as Glo-2, have less obvious repeats. This variable region occurs at the end of the acidic peptide after posttranslational processing, and has been associated with the reduced solubility of oat globulins compared to globulins of other plants [11].

The second more variable sequence region of the oat seed globulins is the C-terminus – which shows considerable variability in both length and sequence (Figure 6). In addition, two of the sequences, Glo-10 and Glo-15, have premature stops in the C-terminus. The sequences in Figure 5 continue the amino acid sequences up to the normal stop codon only to compare to the full-length avenins. The premature stop codons make these two sequences 9 and 17 amino acids shorter than the ancestor sequence before the presumed mutations creating the premature stop codons.

### Relative occurrence of ESTs

Assuming no bias in cloning and sequencing, ESTs can give a measure of the relative transcription of genes. In closely related families such as the seed proteins, the similar sequences argue further against bias. For the two oat major classes of seed proteins, the avenins and globulins, the number of assigned ESTs from cv CDC Dancer is shown in Table 1. For the Glo-17 to Glo-24, only Glo-23 is shown as a representative of this cluster – given problems with assigning ESTs mentioned above.

For the avenins, the number of assigned ESTs follows a bimodal distribution, with avenins 1–3, and 7 having 55–62 ESTs, and avenins 4–6 and 8–9 having 11–23 ESTs. A Chi-square test for randomness of this distribution has a p = 0 at 8 degrees of freedom. For the Glo-1 to Glo-23 globulins, the test results in p<.003 at 15 degrees of freedom.

## Discussion

The current report attempts to derive as complete a set of oat avenin and globulin sequences being transcribed as possible – as seen by forming contigs from the EST set of the oat cultivar CDC Dancer. These results are consistent with previous analyses of oat seed proteins which estimated 11 for oat cv Narmysky 943 [16] and 10 for oat cv Gigant [14] using HPLC and PAGE, but not the estimate of 75 genes using Southern analysis [13].

This contrasts with the report of multiple sequences which used PCR and multiple germplasms in an attempt to correlate specific avenin peptides with celiac immunogenicity [6]. There are several inherent limitations of the latter approach with gene families. The amplified sequences are dependent on designing primers based on known sequences and not the entire gene family – leading to the possibility of missing sequences not matching the primers. In comparing the Real et al. [6] primers to the current set of oat avenin DNA sequences, it is unlikely several of the CDC Dancer sequences would be primed. There is also uncertainty of sequence fidelity, particularly with sequences such as the prolamins and their repetitive nature. In the case of the Real et al. [6] report, four different germplasms yielded 3–8 different sequences – almost certainly only a subset of the entire avenin sequence families from those germplasms. This leads to a difficulty in determining the distribution of celiac peptides and predicting immunogenicity of specific germplasms.

For the globulin genes, a total of 24 distinct sequences were identified for the hexaploid oat cv CDC Dancer. This contrasts with a previous estimate of 150 for globulins for hexaploid *A. sativa* and similar ratios for other *Avena* species [13], but agrees with the 21–30 estimate of Shotwell et al. [11]. Interestingly, it was estimated that there are about 20 oat globulin large subunits and only 10 small subunits using 2D PAGE [8]. It was speculated above that the CDC Dancer globulins without 3′ assigned ESTs was likely due to identical DNA sequence over that region, and the 3′ ESTs from those genes being assigned to another contig due to the inability to distinguish the gene of origin. The 3′ coding regions encode the small subunit, which would also explain why the protein PAGE found fewer small subunits than large; i.e., due to coding identical acid polypeptide amino acid sequences in multiple genes.

Although no genome assignments have been made, evenly distributed genes would indicate about three avenin genes and eight globulin genes per hexaploid oat homoeologous genome. These sequences (genes and encoded polypeptides) were numbered sequentially after amino acid alignments; i.e., Avenin-1, Glo-9, etc. These names are shortened versions (for convenience of reading in the report) of a preferred longer name also denoting the germplasm (Avenin-CDCDancer-1, Glo-CDCDancer-9) where ‘CDCDancer’ is understood and not included as long as there are no other germplasms involved. Future comprehensive analyses of other oat germplasms are recommended to follow similar nomenclature to facilitate comparisons among germplasms.

It has previously been reported that there are more avenin than globulin mRNAs in developing oat seeds [10]. Such a finding is not confirmed in the present report. Initial screening of CDC Dancer ESTs found 887 and 351 ESTs of globulins and avenins, respectively – a ratio of approximately 2.5. On a mass basis, considering the average globulin and avenin sizes (Table 1), the EST numbers suggest mRNAs encoding approximately 6 times as much protein mass for globulins as avenins – assuming equal rates of polypeptide elongation. This is consistent with estimates of the percent of seed protein distributed between the globulins and avenins [2] and would avoid the suggestion that there is differential regulation of transcription and/or translation between the two oat seed protein classes [10].

The distribution of the nine avenin and 24 globulin unique sequences in the hexaploid genome is unknown, but would average three avenin and 8 globulin sequences per genome, assuming an equal distribution. Three classes of sequences would not be included in this total; i.e., exact duplicated genes, pseudogenes, and genes expressed at levels too low to be detected with the available ESTs. A complete catalog of sequences must await a total oat genome sequence, but the described sequences will account for most of the avenin and globulin seed proteins in oat cultivar CDC Dancer, and will provide the most of the functional properties of the seed related to protein content.

### Oat Globulins

The oat globulins have been variously described as 11S [15] and 12S [10] globulins. However, a phylogenetic tree indicates X74740 (59,117 Da) and J05485 (55,846 Da) cluster closely within the oat globulins and are also closely related to Glo-11 (55,841 Da) and Glo-15 (56,575 Da), respectively (Figure 6). The difference in 11S vs 12S nomenclature is both historical and a function of specific techniques used in different studies, but since most reports refer to the oat globulins as 12S it is probably best to use 12S as the standard nomenclature.

Of the 24 distinct globulin sequences found in cv CDC Dancer, eight (Glo-1 to Glo-8) form a branch of the oat globulins not previously observed. The most distinguishing features of this branch include sequence differences (such as positions 237–240, 257–259, and 333–340), the structure of the high-glutamine region, and the sequence and structure of the C-terminus (Figure 5).

The globulin high-glutamine region is similar to the avenin variable regions in having, at least within the same globulin, repeats of discernible simple motifs. Although this feature is not as pronounced as in prolamins, it indicates such repeats can evolve separately in different seed protein classes in addition to the prolamins – which are members of the AAI_LTSS protein superfamily that is unique to higher plants and includes members related to a number of pathologies including celiac disease and allergies. The development of such repeat regions may thus be related more to biological roles and the development and tissue site of synthesis rather than evolutionary history.

This separate history is also indicated since the globulins lack the conserved cysteine core characteristic of the AAI_LTSS superfamily. The CDC Dancer globulins all share five conserved cysteines distributed over the amino acid sequence (Figure 6). In addition, many globulins have a sixth cysteine at position 173 and Glo-9 has a seventh cysteine at position 423. The pattern of intra- and intermolecular disulfide bonds is not known, but the frequency of globulins with an odd number of cysteines raises the question of whether at least some of the globulins can aggregate through disulfide bonds.

It was noted that two of the CDC Dancer globulins have slightly truncated C-termini due to premature stop codons, resulting in polypeptides that would be 9 and 17 amino acids shorter than assumed to have existed prior to DNA mutations that introduced premature stop codons. Such 3′ mutations in other cereal seed protein coding sequences have recently been reported for a barley B-hordein [21] and a wheat γ-gliadin [22]. In those two cases the mutations involved frame shifts that created new stop codons and slightly shorter or longer polypeptides, respectively. Defective mRNAs tend to be removed via a nonsense-mediated-decay (NMD) mechanism [23] that prevents aberrant protein synthesis. These examples from cereal seed proteins may indicate the NMD requires a certain length of aberrant mRNA before acting.

### Oat Avenins

Avenins, as a group, are known to be present as both monomers and in disulfide-linked aggregates [6]. Assuming that CDC Dancer Avenins 1–7 have all eight cysteines (Figure 1) occupied by four intramolecular disulfide bonds (Figure 2) characteristic of many members of the AAI_LTSS superfamily of proteins that form four intramolecular bonds presumably to stabilize the core structure, those seven avenins would exist as monomers in the oat seed. On the other hand, Avenins 8–9 have the ninth cysteine in the final C-terminal position available for external bonds and are likely the aggregating members of the avenin complement – at least for cv CDC Dancer. However, the aggregation would be limited to dimers if the conserved eight cysteines are completely occupied by intramolecular bonds. Larger aggregates would require either some avenins to not completely form the four conserved bonds or the previously reported pattern of bonds is incorrect [13]. This disulfide pattern is different from that reported for wheat γ-gliadins [24] and LMW-glutenins [25]. In particular, the tandem cysteines of positions 145–146 are reported to form a disulfide bond [12] compared to the wheat proteins where the two tandem cysteines form bonds with more distant cysteines within the prolamin. Although disulfide bonds within tandem cysteines are generally considered sterically unfavorable, they have been reported in other proteins and have been referred to as “forbidden” disulfides that may be involved in specific roles such as redox-regulation of proteins [26]. Why related cereal prolamins should have such different disulfide patterns is unknown.

The monomeric and aggregating members of the oat avenin family have recently been termed “gliadin-like avenins” and “glutenin-like avenins”[6]. This unfortunate nomenclature is unnecessary and confusing. Whether or not a prolamin can form intermolecular disulfide links is completely a function of its cysteine residue number and placement and not derived directly from evolutionary relationships. The term “gliadin-like” at least suggests such a direct homologous connection. The more direct “monomeric” and “aggregative” descriptions are to be preferred.

In addition, the use of the term “avenin” has not been carefully employed. It should be reserved to oat proteins that manifest at least the basic defining characteristic of prolamins; i.e., seed proteins high in glutamine plus proline amino acid residues. In addition, to be consistent with the accepted prolamin families of other grasses, the proline plus glutamine content should approach the percentages seen in other grasses. Among the accepted prolamins, those of maize, sorghum, and rice generally have the lowest Q+P content at approximately 25–30%; e.g., CAA32512 (maize), CAA47640 (maize), AAM94311 (sorghum), and AAA50423 (rice). In the Triticeae tribe (wheat, barley, rye), the prolamins can reach over 70% percent glutamine plus proline. In oats, the generally recognized avenins have percents in the 35–50% range, including all the CDC Dancer avenins of the current report (39.4–47.6%). Any oat sequence with a Q+P percent significantly less than prolamins in rice and maize is unlikely to be a genuine avenin and should not be referred to as such. For example, GenBank protein sequence P27919 is annotated as an avenin with some homology to oat globulins [27]. An examination of the sequence by BLAST finds the first half matches exactly to vromindolins, proteins closely related to starch granule associating proteins such as the wheat puroindolins. The second half matches exactly to globulin sequences – leading to speculation that P27919 is almost certainly a chimera created during the cloning process of the encoding mRNA. There are also no plant ESTs that cross the boundary of the two different sequence regions – further supporting a chimeric sequence. In addition, the proline + glutamine content is only 10%. Thus, even without being a chimera, P27919 would not qualify as a prolamin (avenin). Other likely chimeras include entries AGB56859 and AGB56868.

It has also been proposed to group the oat avenins based on their electrophoretic mobility into α, β, γ, and ω classes as originally done for wheat prolamins [9]. However, the amount of DNA sequence data now available more accurately allows evolutionary groupings and is a more accurate reflection of sequence relationships.

In contrast to the single longer repetitive domain found in Triticeae prolamins, the oat avenins contain two shorter high proline + glutamine domains. A vertical alignment of the two repetitive domains from examples of each of the four classes of oat avenins (Figure 4) shows that, as with the Triticeae, the repeats are not exact, but mainly variants of a motif with differences likely caused by single base changes in the genes, and duplication/deletions of either whole or portions of repeats. Even though the two repeat domains are shorter than typically seen with the Triticeae prolamins, the avenin repeat domains are relatively more uniform – suggesting a more recent evolutionary history.

### Relative transcription

In comparing the number of ESTs assigned to unique sequences, for the number of assignments to have comparative transcription value it is necessary that there be no significant difference in post-translation mRNA processing or in the generation of the EST sequences. This condition is commonly not met among populations of different genes as evidenced by different relative ratios using different technology platforms; i.e., EST abundance versus microarrays [28]. One case where EST abundance is more valid is comparisons within a closely related sequence family expressed at the same developmental stage in the same tissue - such as with the oat avenins and globulins. Within each class, the sequences are similar enough to reduce the likelihood of differential effects during EST generation. Assuming that the current analysis’ apparent bi-modal distribution of avenin ESTs assigned to specific sequences is valid, there are two possible explanations. One is that there is differential transcription and/or mRNA processing within the avenin genes. The second possible explanation is that the sequences with the highest number of ESTs are the result of multiple genes encoding identical sequences. The latter case could not be detected by EST analysis and could even be difficult when whole-genome sequences are available since exact gene duplications tend to collapse into fewer apparent sequences during genome assemblies. Thus, for example, in the present case it cannot be ruled out that the Avenin-1 assigned ESTs come from four identical genes compared to one gene for Avenin-4 (Table 1). In either case, the ESTs indicate a differential distribution of ESTs and potentially of mRNAs from which the ESTs were generated. The assigned EST distribution within the globulins had a similar apparent two classes (full-length, not including the cluster with Glo-23), with Glo-1,4,7,8,14,15 averaging 15 ESTs and the remaining 9 globulins averaging 35 ESTs.

Another limitation of an EST analysis is that only genes being actively transcribed at sufficient levels will be detected. In the case of the oat seed proteins, no information can be obtained on pseudogenes such as has been reported for an oat globulin (X68648), nor can it detect genes expressed at such low levels that no EST contigs can be assembled. Neither case affects the major spectrum of ESTs representing mRNAs available for polypeptide synthesis or has any functional implication for the spectrum of avenin and globulin proteins in oat seeds.

## Conclusion

Although oats are purported to be safe for celiac patients [29], lacking has been a complete description of the oat seed proteins in at least one specific germplasm, and then available for epitope analysis and to serve as a baseline for comparing different oat cultivars. A comparison of oat cultivars for immunogenicity for patients with celiac disease found a range of reactivity [30]. Such studies offer the opportunity to associated celiac reactivity with specific sets of seed proteins. The increasing efficiency of DNA sequencing could be used to perform deep ESTs on oat seed from such sets of germplasms. If specific sequences and epitopes could be associated with levels of celiac response, the ESTs could serve as a complement or substitute for immunological or clinical initial evaluations of oat cultivars.

## Supporting Information

Figure S1 **Phylogenetic tree of avenins.** A phylogenetic tree was generated with the Neighbor-joining algorithm from a ClustalW alignment of CDC Dancer avenin amino acid sequences and the sequences of all available full-length oat avenin entries in Genbank. CDC Dancer avenins are in bold; e.g., Av-1.
(PDF)

Table S1 **Contig ESTs. Listed are the ESTs forming all CDC Dancer avenin and globulin contigs.**
(XLS)

File S1 **CDC Dancer avenin DNA sequences in fasta format.**
(TXT)

File S2 **CDC Dancer avenin derived amino acid sequences in fasta format.**
(TXT)

File S3 **CDC Dancer globulin DNA coding sequences in fasta format.**
(TXT)

File S4 **CDC Dancer globulin amino acid sequences in fasta format.**
(TXT)
