# Supplementary figures and images for: The Spectrum of Major Seed Storage Genes and Proteins in Oats (Avena sativa)
Source: PLoS One. 2014 Jul 23;9(7):e83569. doi: 10.1371/journal.pone.0083569 (PMC4108316; doi:10.1371/journal.pone.0083569)

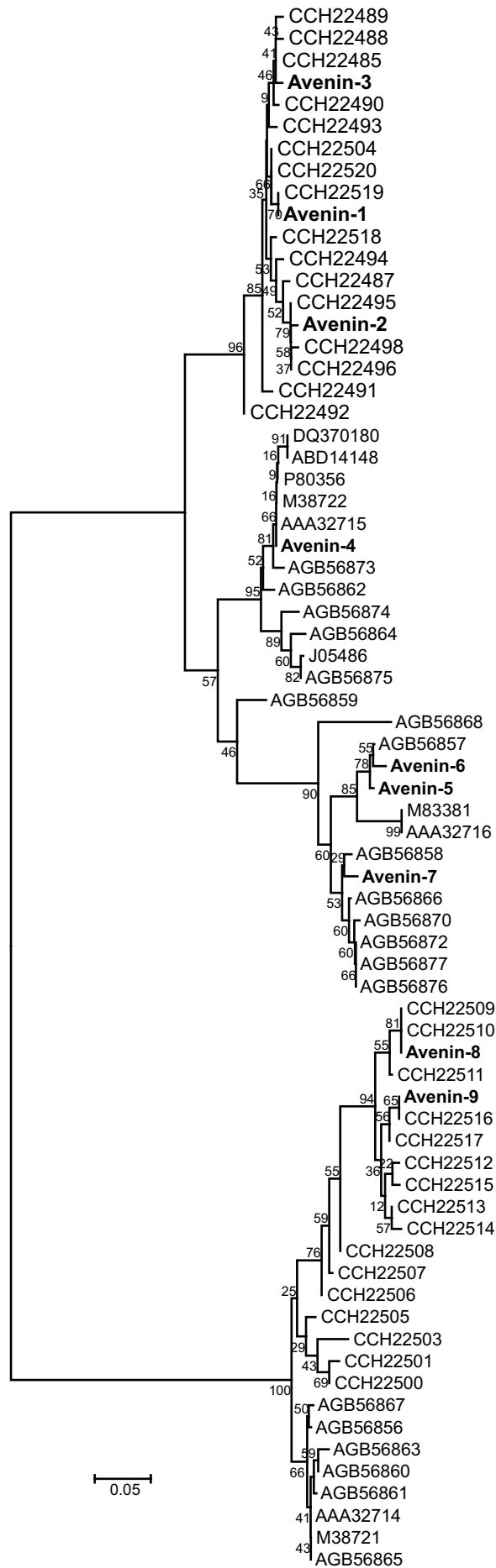

Supplement: Figure S1 — Phylogenetic tree of avenins. A phylogenetic tree was generated with the Neighbor-joining algorithm from a ClustalW alignment of CDC Dancer avenin amino acid sequences and the sequences of all available full-length oat avenin entries in Genbank. CDC Dancer avenins are in bold; e.g., Av-1. (PDF) [file pone.0083569.s001.pdf]
